# Basic Cells Special Features and Their Influence on Global Transport Properties of Long Periodic Structures

**DOI:** 10.3390/e26110942

**Published:** 2024-11-03

**Authors:** Luna R. N. Oliveira, Marcos G. E. da Luz

**Affiliations:** Departamento de Física, Universidade Federal do Paraná, Curitiba 81531-980, PR, Brazil; lunaoliveira@ufpr.br

**Keywords:** transmission properties, localized potentials, scattering amplitudes, band structure

## Abstract

In this contribution, we address quantum transport in long periodic arrays whose basic cells, localized potentials U(x), display certain particular features. We investigate under which conditions these “local” special characteristics can influence the tunneling behavior through the full structure. As the building blocks, we consider two types of U(x)s: combinations of either Pöschl–Teller, $U_{0}/\cosh^{2}\left[\alpha \;x\right]$, potentials (for which the reflection and transmission coefficients are known analytically) or Gaussian-shaped potentials. For the latter, we employ an improved potential slicing procedure using basic barriers, like rectangular, triangular and trapezoidal, to approximate U(x) and thus obtain its scattering amplitudes. By means of a recently derived method, we discuss scattering along lattices composed of a number, *N*, of these U(x)s. We find that near-resonance energies of an isolated U(x) do impact the corresponding energy bands in the limit of very large *N*s, but only when the cell is spatially asymmetric. Then, there is a very narrow opening (defect or rip) in the system conduction quasi-band, corresponding to the energy of the U(x) quasi-state. Also, for specific $U_{0}$’s of a single Pöschl–Teller well, one has 100% transmission for any incident E>0. For the U(x) parameters rather close to such a condition, the associated array leads to a kind of “reflection comb” for large *N*s; $|T_{N}{\left(k\right)|}^{2}$ is not close to one only at very specific values of *k*, when $|T_{N}{|}^{2}\approx 0$. Finally, the examples here—illustrating how the anomalous transport comportment in finite but long lattices can be inherited from certain singular aspects of the U(x)s—are briefly discussed in the context of known effects in the literature, notably for lattices with asymmetric cells.

## 1. Introduction

Regular and repetitive structures are common in the natural world [1,2,3,4], arising from uncountable phenomena [5]. So, often the associated patterns are used to probe, and thus to understand, a vast number of distinct processes [6,7]. Potentially, their organization and related general—in the sense of recurrent—symmetries should determine usual traits and properties [6,8], resulting in rather universal features. In fact, the global characteristics of energy bands in arbitrary crystals, certain cyclical attributes of the natural elements (even allowing the construction of a periodic table) and the replicating trends in biology [8] are all good examples of such an ubiquity.

Fitting perfectly well into the above description is the relevant problem of wave (either elastic, electromagnetic or matter) scattering in periodic lattices [9,10,11]. Nonetheless, there are situations in which the dynamics in determined orderly networks can display unusual comportment. In some instances, this is due to uniquely engineered systems [12,13,14]. One illustration, which is a bit controversial, is the emergence of classical-like chaotic behavior due to quantum scattering in Fibonacci lattices (for a historical account and review of the original literature, see [15]). Another relates to particular (quasi-transparent) scattering states, which are connected to the non-trivial zeros of the Riemann zeta function (and so, with the Riemann’s hypothesis) in the so-called logarithmic chains; refer to [16] and refs. therein.

But anomalous or unusual effects do not appear only when waves are propagating in a too-tailored structure. They can also take place in typical lattices, as nicely discussed and exemplified in refs. [17,18,19,20]. However, there may be a caveat. The mentioned effects might be (theoretically) unveiled and explained only if one considers large enough lattices, when multiple scattering [21] becomes fundamental and is treated exactly or at least in the proper orders of approximation [22,23,24].

Based on a well-established Green’s function approach [25] (for a review, see [26]), very recently a method has been developed [27] to calculate the scattering properties of arbitrarily long lattices formed by *N* equally spaced building blocks (or cells). The framework fully includes multiple scattering processes, and the final exact expressions are simply dependent on the basic cell reflection, *R*, and transmission, *T*, coefficients. Therefore, the results are exact if *R* and *T* are exact. Moreover, for *N* extremely large—e.g., numerically *N* has been analyzed up to $10^{10}$ in [27]—one recovers most of the essential features of the energy bands corresponding to the infinite lattice case.

Employing the above-mentioned protocol, in this contribution we shall discuss the transmission properties across long regular lattices assuming special building blocks. We suppose that symmetric and asymmetric basic cells display particular characteristics. Then, we examine if these “local” features can introduce additional trends in the transport behavior of the whole structure. In general, we find that the near-resonance energies of the cell influence some aspects of the band’s shape, but only when the cells are asymmetric. In this case, we observe a narrow gap opening, a rip, in the band corresponding to the energy of the quasi-state of the cell. This effect, nevertheless, becomes noticeable only for relatively large *N*s, i.e., large structures.

**Figure 1:**
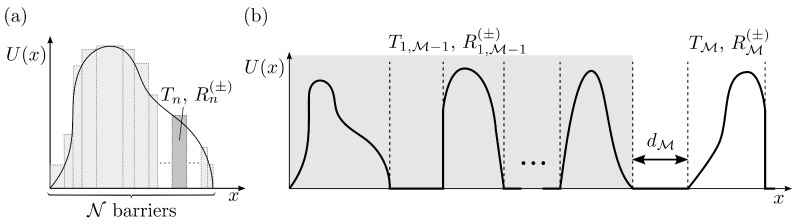
(**a**) Schematics of a continuous localized potential approximated by a set of N elementary (here rectangular) barriers. The darker gray potential indicates the *n*-th barrier, with its reflection and transmission coefficients shown. (**b**) An array of M arbitrary localized, compact support potentials.

To construct such special cells, we consider two instances, both relying on localized *U*s. The difference is that, in the first, for Pöschl–Teller (second, for Gaussian) potentials, *R* and *T* are (are not) known analytically. For the latter, we observe that an usual procedure is to approximate a continuous localized U(x) by a collection of N very narrow barriers, often rectangular ones (see Figure 1a). The shape of U(x) is then reproduced by properly setting the barrier heights. Thus, one can employ distinct techniques, such as the transfer matrix method [28], to compute (in general numerically) $T_{U}$ and $R_{U}$. But not uncommonly, N must be large, especially when U(x) is a peak-shaped function. We show that the usage of basic triangular and trapezoidal (besides rectangular) barriers, whose scattering coefficients are analytically accessible, can considerably reduce N. Such basic potentials form a composed building block (CBB), allowing good analytic approximations for $T_{U}$ and $R_{U}$. Finally, once we have the cell scattering amplitudes, we can study arrays of *N* equally spaced U(x)s via the method in [27].

The paper is organized as follows. We present a very brief overview of the general approach in [27] and its basic equations in Section 2. We exemplify how to approximate a continuous localized potential by a collection of barriers in Section 3. We present some properties of long lattices in Section 4. We explore anomalous behavior in the transmission probability originated from asymmetric cells in Section 5. Final remarks and conclusions are drawn in Section 6. More technical necessary results are provided in the Appendices.

## 2. Brief Review on the Approach Used to Study Transport in 1D Periodic Structures

Consider an arbitrary system composed of M non-overlapping compact support potentials, $U_{m}$. So, each $U_{m}$ (m=1,⋯,M) is non-null only within a finite spatial interval. The resulting structure is illustrated in Figure 1b. Further, assume that the scattering coefficients of $U_{m}$ are given by $T_{m}$ and $R_{m}^{\left(\pm \right)}$. Here, these coefficients are written up to the phases usually associated with either the width (transmission) or the end positions of $U_{m}$ (for details, see Appendix A). The superscript (±) identifies the incident direction for a wave incoming from x→∓∞. Obviously, if the potential is symmetric, $R_{m}^{\left(+\right)}=R_{m}^{\left(-\right)}=R_{m}$.

For the separation between potentials m=M−1 and m=M being denoted as $d_{M}$, Figure 1b, the reflection, $R_{1,M}^{\left(\pm \right)}$, and transmission, $T_{1,M}$, coefficients for the whole array formed by $U_{1},U_{2},\ldots ,U_{M}$ can be calculated from that formed by $U_{1},U_{2},\ldots ,U_{M-1}$ through the recurrence relations

(1) $$\begin{array}{ccc} R_{1,M}^{\left(+\right)} & = & R_{1,M-1}^{\left(+\right)}+\frac{R_{M}^{\left(+\right)}\;T_{1,M-1}^{2}\;\exp\left[2\;i\;k\;d_{M}\right]}{1-R_{1,M-1}^{\left(-\right)}\;R_{M}^{\left(+\right)}\;\exp\left[2\;i\;k\;d_{M}\right]},\\ R_{1,M}^{\left(-\right)} & = & R_{M}^{\left(-\right)}+\frac{R_{1,M-1}^{\left(-\right)}\;T_{M}^{2}\exp\left[2\;i\;k\;d_{M}\right]}{1-R_{1,M-1}^{\left(-\right)}\;R_{M}^{\left(+\right)}\;\exp\left[2\;i\;k\;d_{M}\right]},\\ T_{1,M} & = & \frac{T_{1,M-1}^{\left(+\right)}\;T_{M}^{\left(+\right)}\;\exp\left[i\;k\;d_{M}\right]}{1-R_{1,M-1}^{\left(-\right)}\;R_{M}^{\left(+\right)}\;\exp\left[2\;i\;k\;d_{M}\right]}. \end{array}$$

Such expressions follow directly from the exact Green’s function method in [25].

Now, suppose a periodic finite lattice with *N* identical localized potentials, U(x), where any two neighbor *U*s are separated by *L*. In this case, we can simplify the notation by writing $T_{1,M}=T_{N}$ and $R_{1,M}^{\left(\pm \right)}=R_{N}^{\left(\pm \right)}$ and the scattering amplitudes of *U* by $R^{\left(\pm \right)}$ and *T*. Moreover, for any M we have $d_{M}=L$. Then, it has been shown [27] that the above recurrence relations lead to (using the fact that always $|R^{\left(+\right)}{|}^{2}=|R^{\left(-\right)}{|}^{2}={\left|R\right|}^{2}$)

(2) $$|T_{N}{|}^{2}=|1-\frac{C_{N}}{C_{N-1}}|\;\frac{{\left|T\right|}^{2}}{{\left|R\right|}^{2}},\;{\left|R_{N}^{\left(\pm \right)}\right|}^{2}=|1-\frac{C_{N}}{C_{0}}{|}^{2}\;\frac{1}{{\left|R\right|}^{2}},$$

where

(3) $$C_{N}=\frac{\left(1+C_{0}\;\Gamma_{-}\right)\;{\left(\Gamma_{-}/\Gamma_{+}\right)}^{N}-\left(1+C_{0}\;\Gamma_{+}\right)}{\left(C_{0}-\Gamma_{+}\right)\;{\left(\Gamma_{-}/\Gamma_{+}\right)}^{N}-\left(C_{0}-\Gamma_{-}\right)}$$

and

(4) $$\gamma =\frac{1+\left(T^{2}-R^{\left(+\right)}R^{\left(-\right)}\right)\;\exp\left[2\;i\;k\;L\right]}{i\;T\;\exp[i\;k\;L]},\;\Gamma_{\pm }=\frac{\gamma \pm \sqrt{\gamma^{2}+4}}{2}.$$

The above expressions are exact and have no limitations regarding the values of *N*. So, the transmission and reflection probabilities for extremely long arrays are easy to obtain from these ready-to-use formula.

### Approximating a Rapidly Decaying Potential Through Compact Support Potentials

Suppose a continuous quickly decaying potential, U(x), i.e., it rapidly tends to zero as |x| increases. In principle, we can approximate U(x) as a block of juxtaposed N barriers, as illustrated in Figure 1a. Then, from our present framework, the scattering coefficients of U(x), $T_{U}$ and $R_{U}^{\left(\pm \right)}$ can be computed from $T_{1,M}$ and $R_{1,M}^{\left(\pm \right)}$ in Equation (1), with M=N and all *d*s set to zero.

We should remark that, in the potential slicing technique, one often assumes a relatively large set of rectangular barriers to approximate U(x) and then employs the transfer matrix method for the calculations (for a didactic review, see, e.g., Ref. [29]). However, this scheme is considerably simplified and computationally speed up if we allow a more diverse set of barriers, namely, not only rectangular (r) but also triangular (t) and trapezoidal ($\tilde{t}$) shapes, all presenting exact analytical scattering coefficients; see Appendix B. This drastically reduces N, also making Equation (1) simple to handle.

## 3. Certain Special U(x)s and Their CBBs Description

For the sake of nomenclature, we will refer to the collection of N localized potentials closely describing a certain *U* as the *composed* building block (CBB) representing U(x). So, the goal is to have $T_{U}$ and $R_{U}^{\left(\pm \right)}$ well approximated by the corresponding $T_{CBB}=T_{1,N}$ and $R_{CBB}^{\left(\pm \right)}=R_{1,N}^{\left(\pm \right)}$. Further, unless otherwise explicitly mentioned, we will suppose m=ℏ=1, with m being the particle mass. Accordingly, the energy is given by $E=k^{2}\hbar^{2}/2m=k^{2}/2$.

### 3.1. The Gaussian Potential

**Figure 2:**
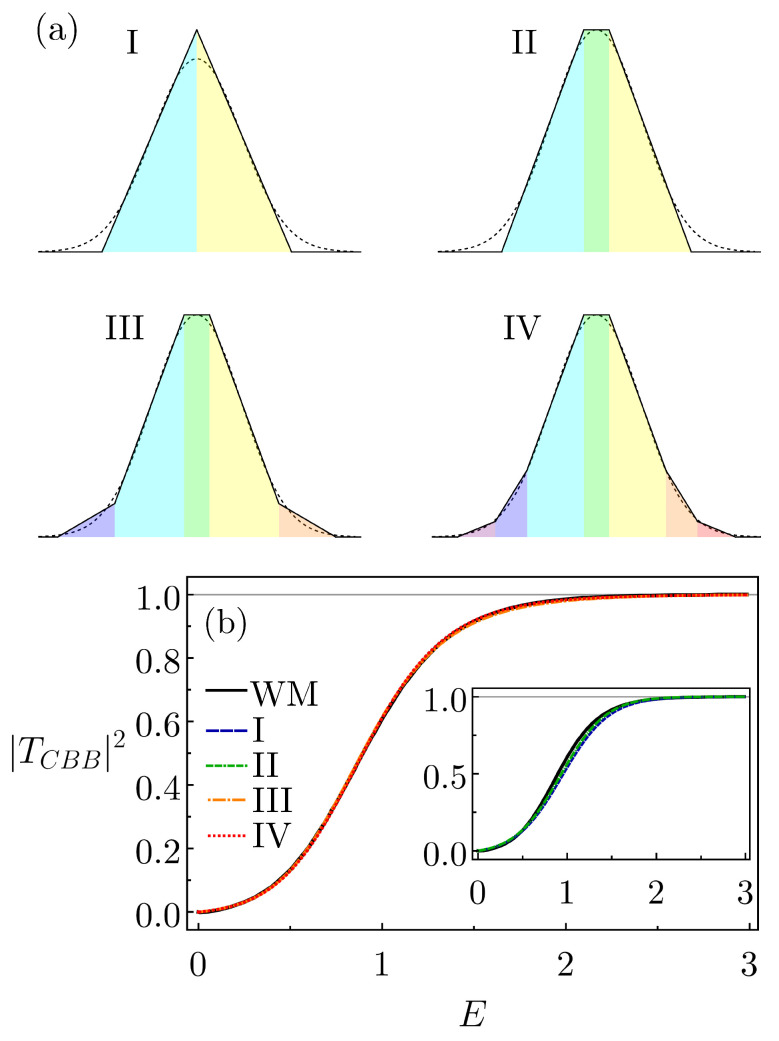
(**a**) Distinct CBBs that approximate a Gaussian potential: CBB-tt (I), CBB-trt (II), CBB-t$\tilde{t}$r$\tilde{t}$t (III) and CBB-t$\tilde{t}$$\tilde{t}$r$\tilde{t}$$\tilde{t}$t (IV). Here, CBB-abc… means that the CBB is formed, from left to right, by the sequence a, b, c, … of juxtaposed barriers; moreover, t, $\tilde{t}$ and r stand, respectively, for triangular, trapezoidal and rectangular shapes. The parameters are those in Table 1. (**b**) As a function of incident energy, the $|T_{CBB}{|}^{2}$s of CBBs III and IV are compared with the Gaussian potential actual transmission probability, calculated with the Wronskian method (WM) in Ref. [30,31]. The agreement is very good for both CBBs. In the inset is the same type of comparison, but for CBBs I and II.

First, we discuss the Gaussian potential

(5) $$U_{G}\left(x\right)=U_{0}\exp\;\left[-\frac{{\left(x-\mu \right)}^{2}}{2\;\sigma^{2}}\right],$$

for which there are no known analytic expressions for the reflection and transmission amplitudes. We approximate $U_{G}$ in four different ways, considering four sets of CBBs: triangle–triangle (I), triangle–rectangle–triangle (II), triangle–trapezoid–rectangle–trapezoid–triangle (III) and triangle–trapezoidal–trapezoid–rectangle–trapezoid–trapezoid–triangle (IV). The constructions are depicted in Figure 2a. To estimate how similar the CBB structures are to the original Gaussian potential, we can compute the difference in the areas. For $U_{G}$ with the parameters μ=0, $\sigma =\sqrt{2}/2$, $U_{0}=1$ and for CBBs with the parameters given in Table 1, the areas of CBB-I, CBB-II, CBB-III and CBB-IV differ from that of $U_{G}$, respectively, by 8.11%, 6.48%, 3.50% and 2.56%. The parameters in Table 1 (and in the next table) were manually adjusted (in trial-and-error attempts), seeking to minimize the areas’ differences. Of course, automatized numerical procedures would improve the agreement even more. We intend to implement algorithms to do so in a future contribution.

As previously mentioned, we obtain $T_{CBB-I,II,III,IV}$ from the recurrence relations in Equation (1) and from the basic shapes of individual *R*s and *T*s given in Appendix B (the resulting final analytic expressions are a bit lengthy and are thus not shown here, but they are easy to derive and handle from any algebraic software). In Figure 2b, we compare the transmission probabilities for these CBBs with that of the actual Gaussian calculated via the numerically accurate Wronskian method of Ref. [30,31]. From a direct visual inspection, one realizes that the agreement is very good for CBB-III and CBB-IV and fairly good for CBB-I and CBB-II. As an extra test, we have integrated the curves ${\left|T\left(E\right)\right|}^{2}$ in the energy region shown in Figure 2b. The difference between the results for the Gaussian and the distinct CBB-I,II,III,IV are, respectively, 2.59%, 1.59%, 0.43%, 0.36%. These small values, especially for the latter two, quantitatively confirm the observed, rather fine, concordance.

### 3.2. The Pöschl–Teller Potential

Next, we address the Pöschl–Teller potential,

(6) $$U_{PT}\left(x\right)=\frac{U_{0}}{\cosh^{2}\left[\alpha \left(x-c\right)\right]},$$

which recently has found many applications in condensed matter physics and quantum chemistry [32,33,34,35,36]. The exact quantum scattering amplitudes for $U_{PT}$ are presented in Appendix C. For a full analysis of $U_{PT}$ in terms of a Green’s function approach—the key technique used in [27] to derive the expressions in Section 2—refer to Ref. [37].

**Figure 3:**
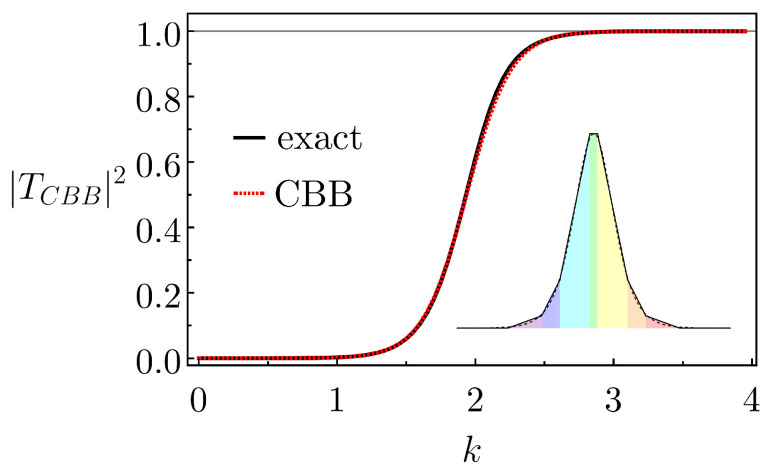
The exact probability transmission versus *k* for the Pöschl–Teller potential compared with that of the CBB (whose composition is shown in the inset). For the parameter values, see main text.

Previously, seven elementary barriers were enough to yield a very good approximation for the Gaussian potential (CBB-IV in Figure 2a). So, we again use this same number to construct the CBB for the Pöschl–Teller potential. The resulting CBB, of concrete configuration t$\tilde{t}$$\tilde{t}$r$\tilde{t}$$\tilde{t}$t, is depicted in the inset of Figure 3. Setting c=0, α=1 and $U_{0}=2$, the corresponding parameters for the CBB’s first t$\tilde{t}$$\tilde{t}$r basic shapes are (by symmetry, those for the last three barriers, namely, $\tilde{t}$$\tilde{t}$t, are akin) w=1.30,0.70,1.15,0.3, $U_{a}=0,0.11,0.47$, $U_{b}=0.11,0.47,2$ and $\tilde{U}=2$. We notice that the areas of this CBB and of the actual $U_{PT}\left(x\right)$ differ by only 2.92%. Figure 3 compares the transmission probability for the CBB and for the $U_{PT}$ (this latter obtained from the exact expression in Equation (A7)). As expected, the curves are very similar, e.g., when integrated, differing by only 0.36%.

## 4. Simple Finite Periodic Lattices

We shall now consider “simple” finite periodic lattices. By simple we mean arrays of *N* equally spaced cells, U(x)s. For these cells, we assume a single symmetric $U_{G}$ or $U_{PT}$. Also, the distance between two successive *U*s are such that the potentials have a fairly negligible overlap (for the curious scattering properties of two highly superposed Pöschl–Teller potentials, see [37]).

### 4.1. The Gaussian Case

**Figure 4:**
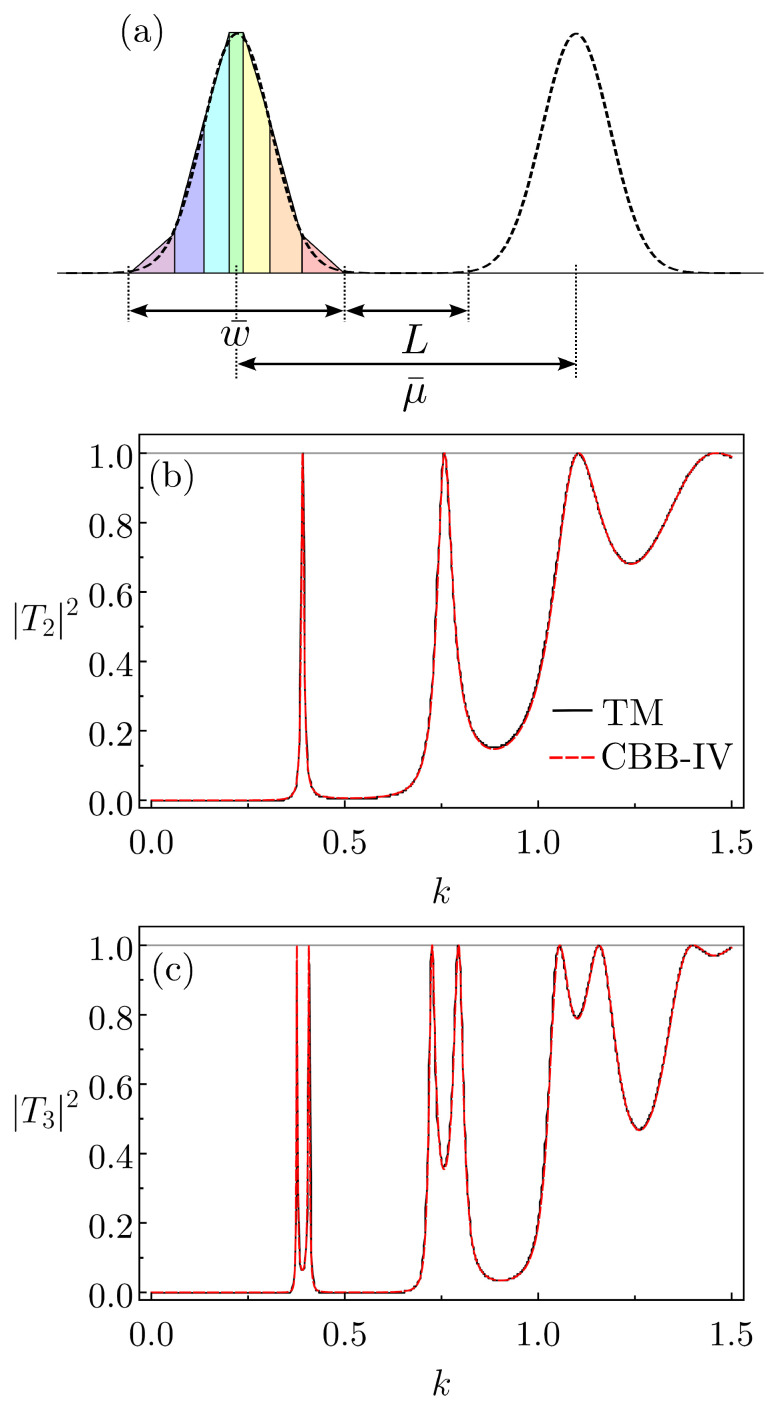
(**a**) Schematics of two successive Gaussians (dashed curves) belonging to an array of *N* localized barriers. As indicated, each Gaussian is approximated by a proper CBB-IV. The full transmission probability versus *k* for the case of (**b**) N=2 and (**c**) N=3, calculated from the present approach using the CBB-IV and from the transfer matrix method (TM) in [38] (the corresponding curves have been digitalized directly from Ref. [38]). Each Gaussian reads $U_{G}\left(x\right)=\exp\left[-x^{2}\right]$; in addition, $\bar{\mu }=8$. For the first four BBs of the CBB-IV, namely t$\tilde{t}$$\tilde{t}$r, the parameters are w=0.6,0.5,0.9,0.4, $U_{a}=0,0.06,0.3$, $U_{b}=0.06,0.3,1$ and $\tilde{U}=1$. To align with Ref. [38], specifically for this example, we set $2\mu /\hbar^{2}=1$.

For the Gaussian arrays, we set a distance, $\bar{\mu }$, between the cells. As the transmission and reflection amplitudes of $U_{G}$, we shall consider those of the corresponding CBB-IV. Hence, for $\bar{w}$ denoting the base length of the whole CBB-IV structure, as illustrated in Figure 4a, we define the lattice parameter as $L=\bar{\mu }-\bar{w}$.

Naturally, we should check if by using CBB-IV, instead of the actual $R_{G}$ and $T_{G}$, we can still obtain a reliable description for the full lattice transport properties. So, as a verification, we compare $|T_{N}{\left(k\right)|}^{2}$ from the expression in Eqution (2), setting $R=R_{CBB-IV}$ and $T=T_{CBB-IV}$, with numerical schemes. For N=2 and N=3, the traditional slicing method (based on the transfer matrix procedure) employing N=88 rectangular barriers for each Gaussian has been implemented in [38], leading to very accurate results. Figure 4b,c show plots for the transmission probability as a function of *k*. As one can see, the agreement between the two approaches is very good, but in our case computationally rather inexpensive. In this way, in the following we analyze a lattice with $N=10^{4}$ Gaussians, a configuration that, to the best of our knowledge, has not been addressed previously in the literature for this type of cell.

We further remark that while the present analysis might be feasible using other frameworks, such as the transfer or scattering matrix approaches (see, e.g., [39,40,41]), the current method is computationally more efficient. This fact stems from Equation (3), where the number of cells only enters as an exponent of certain quantities, instead of representing the number of matrices to be multiplied (for more details, see [27]). Also, although we treat the Gaussian potential as a CBB, we emphasize that the general expressions for the transport along the full lattice are exact and do not rely on iterative procedures.

**Figure 5:**
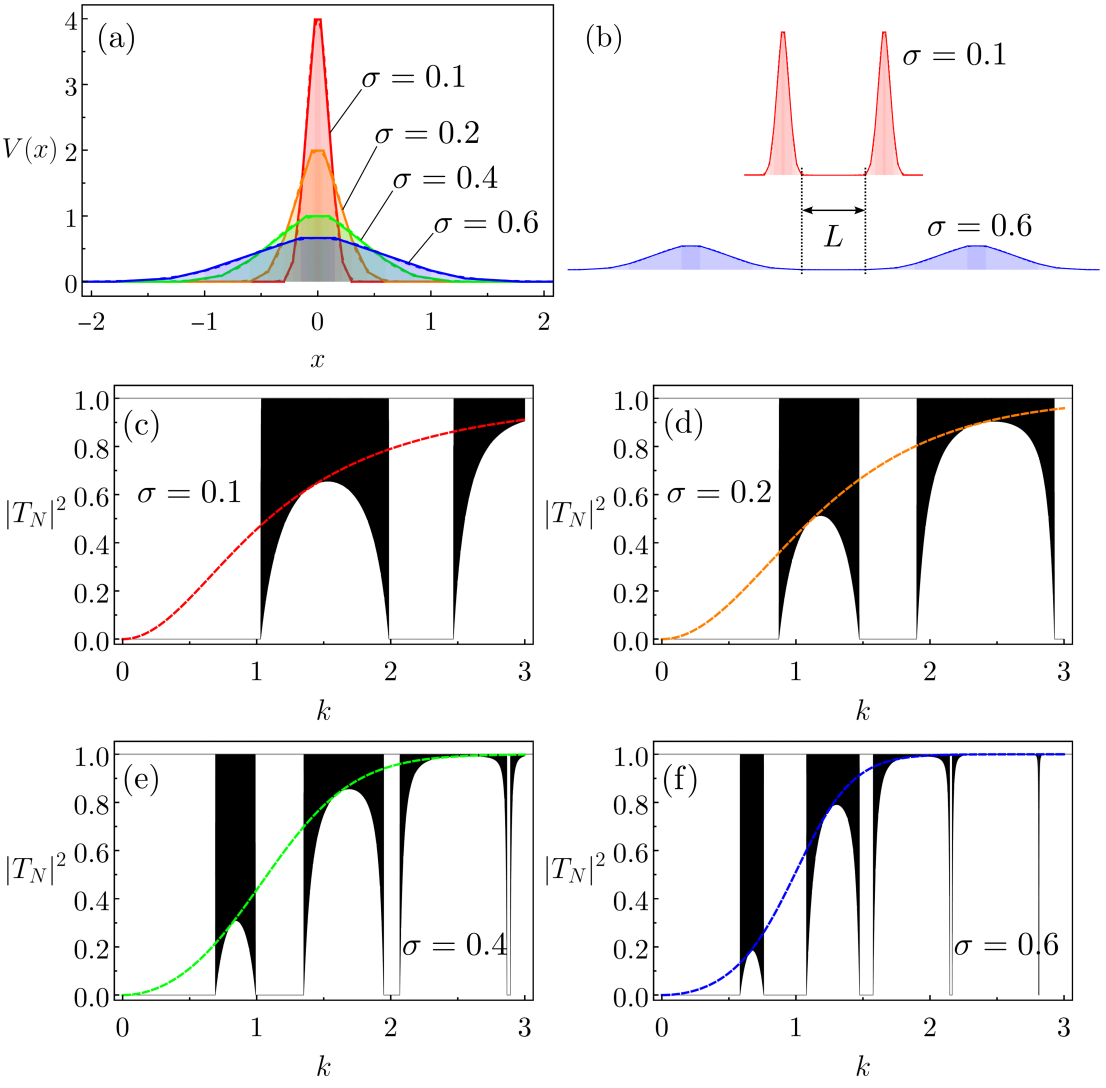
(**a**) The four CBB-IVs that model the continuous Gaussian potentials, forming arrays of $N=10^{4}$ barriers. (**b**) Illustration of two successive Gaussians composing a lattice in the cases of σ=0.1 and σ=0.6. The parameters are always chosen such that $L=\bar{\mu }-\bar{w}=1$. Transmission probabilities as a function of *k* for the arrays with (**c**) σ=0.1, (**d**) σ=0.2, (**e**) σ=0.4, (**f**) σ=0.6. The dashed curves represent the transmission probability for the corresponding single CBB-IV.

For our finite lattices of $N=10^{4}$ equal Gaussian barriers, we discuss four distinct configurations. The corresponding cells display the following parameters pairs $\left(U_{0},\sigma \right)$; see Equation (5): (4, 0.1), (2, 0.2), (1, 0.4), (0.66, 0.6). Then, the cells of the four cases have the same area. The CBB-IVs used to model the Gaussian barriers are depicted in Figure 5a, with the parameters listed in Table 2. The distances between successive barriers, $\bar{\mu }$, are chosen, such that L=1; see Figure 5b. The resulting transmission probabilities as a function of *k* for the distinct arrays are shown in Figure 5c–f. The associated individual $|T_{CBB-IV}{\left(k\right)|}^{2}$ is also shown. Since the areas of the different Gaussians are equal and always L=1, effectively we have a direct relation between $U_{0}$ and σ, and thus we can assume $U_{0}$ as the only free parameter. Hence, in Figure 5c–f the variation of $U_{0}$ is simply equivalent to re-scaling the *k*s, explaining why the quasi-band structures in the four instances are similar, only with distinct widths. One way to picture this re-scaling is to draw a parallel with a tight-binding model. In such a case, the “potential strength” (independent on area) is proportional to the effective hopping integral, which, in turn, is proportional to the band width [42,43]. So, as σ decreases, the band widths increase accordingly. Further, notice that for an array of Gaussians the function $|T_{CBB-IV}{\left(k\right)|}^{2}$ acts as the envelope of the centers of the allowed quasi-bands [44]. This complies with similar features of lattices formed by delta and triangular barriers [27].

We lastly remark that certain works addressing semiconductor superlattices assume Gaussian profiles [45,46,47,48,49]. For instance, for the Gaussians being approximated by rectangular barriers, a photovoltaic device in which a Gaussian superlattice is inserted into a GaAs solar cell has been investigated [50]. Similar constructions have been considered in the analysis of the electronic properties of graphene-based superlattices [51] as well as of phonon tunneling in semiconductor heterostructures [52]. Our present approach would be a very valuable tool for all these problems, permitting one to treat a much larger number of Gaussian potentials, eventually leading to a more realistic description of the aforementioned devices.

### 4.2. The Pöschl–Teller Case

Differently from Section 4.1, for $U_{PT}$ the scattering coefficients are known analytically; see Appendix C. However, there is a caveat. The Pöschl–Teller is a rapidly decaying but not a compact support potential. Therefore, to be able to use the results of Section 2, a few simple modifications are necessary. In fact, how to proceed in such a context has been fully discussed in Ref. [37]. The first change is to introduce extra phases for $R^{\left(\pm \right)}$ and *T*, and the second is to consider a *k*-dependent *L*, both to be implemented in the auxiliary Equation (4).

In particular, the Pöschl–Teller is one of the potentials investigated in detail in [37]. So, here we just use the expressions derived in [37]. For L→L(k) in Equation (4), we have $U_{PT}\left(x\right)=U_{0}/\cosh^{2}\left[\alpha \left(x-c\right)\right]$ (with $\epsilon =U_{0}/E=2U_{0}/k^{2}$), and

(7) $$L\left(k\right)=\frac{1}{\alpha }\left\{\begin{array}{cc} 2I(\alpha c)-\ln[|\epsilon -1|], & \epsilon \neq 1,\\ 2\ln[\cosh[\alpha c]], & \epsilon =1, \end{array}\right.$$

where

(8) $$I\left(\xi \right)=\ln\left[\sinh\left[\xi \right]+\sqrt{\cosh^{2}\left[\xi \right]-\epsilon }\right]+\frac{\sqrt{\epsilon }}{4}\ln\left[{\left(\frac{\sqrt{\epsilon }\sinh\left[\xi \right]-\sqrt{\cosh^{2}\left[\xi \right]-\epsilon }}{\sqrt{\epsilon }\sinh\left[\xi \right]+\sqrt{\cosh^{2}\left[\xi \right]-\epsilon }}\right)}^{2}\right].$$

The above L(k) implies that the width between the centers of two consecutive $U=U_{PT}$ in the lattice is 2c and is thus akin to the distance $\bar{\mu }$ depicted in Figure 4a for the Gaussians. The extra phases multiplying the transmission and reflection coefficients in Equation (4) are given in Appendix C.

**Figure 6:**
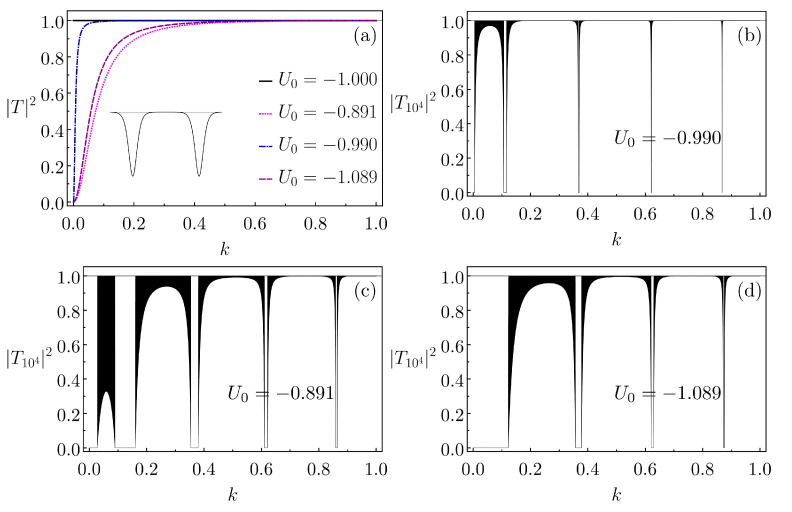
(**a**) Transmission probability as a function of *k* for a single Pöschl–Teller well with α=1 and $U_{0}$ equal to −1.000, −0.891, −0.990 and −1.089. For the three latter cases, being the basic cells of finite periodic lattices with $N=10^{4}$ and 2c=14 (which is the separation between the centers of two successive wells; inset in (**a**)), the corresponding $|T_{N}{|}^{2}$ versus *k* plots are presented in (**b**–**d**).

Interestingly, when $U_{0}<0$ and $1+8m|U_{0}|/\hbar^{2}\alpha^{2}={\left(2n+1\right)}^{2}$ for n=0,1,2,…, there is full transmission across the Pöschl–Teller well for any incident k>0. For example, for $U_{0}=-1$ and α=1, Figure 6a shows that the transmission probability is always one. Likewise, one would obtain 100% transmission along a whole array of these wells, regardless their number, *N*. Figure 6a also displays $|T_{PT}{|}^{2}$ for single wells with the same α, but for depths that are slightly different from −1, namely, $U_{0}=-0.99$ (1% shallower), $U_{0}=-1.089$ (10% deeper) and $U_{0}=-0.891$ (10% shallower). As expected, the further the $|U_{0}|$s are from the mentioned special values, the larger the necessary onset for *k* to make $|T_{PT}{\left(k\right)|}^{2}\approx 1$.

For the three cases with $U_{0}\neq -1$ in Figure 6a, we suppose finite periodic lattices with $N=10^{4}$ and the separation between two successive wells equal to 2c=14 (inset of Figure 6a). The resulting $|T_{N}{|}^{2}$ are shown in Figure 6b–d. We point out that as $U_{0}$ approaches −1, the forbidden quasi-bands tend to become comparatively narrower.

**Figure 7:**
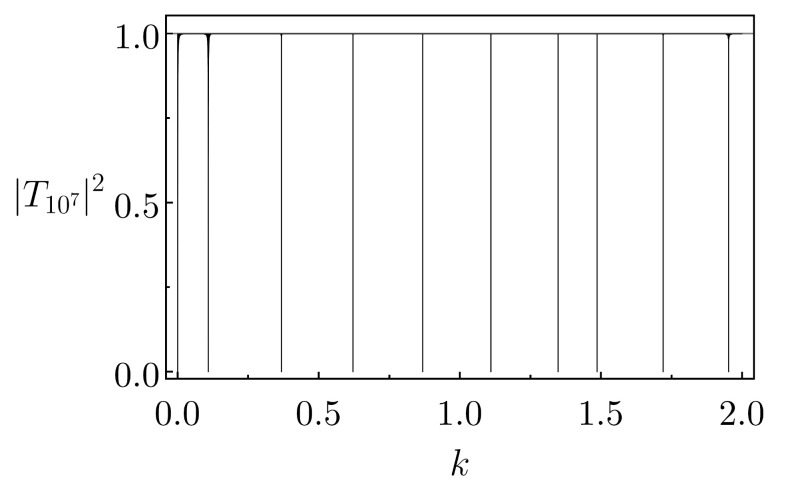
Similar to Figure 6b, but for $U_{0}=-0.999$ and $N=10^{7}$. The extremely narrow (basically spikes) forbidden quasi-bands occur around the wavenumbers 0.109, 0.368, 0.621, 0.868, 1.11, 1.35, 1.49, 1.72, 1.95. Apart from for the spikes, there are almost no fluctuations from $|T_{N}{|}^{2}\approx 1$.

To better appreciate this last behavior, we present in Figure 7 the plot of $|T_{N}{|}^{2}$ versus *k* for an extremely long lattice of $N=10^{7}$ Pöschl–Teller wells with $U_{0}=-0.999$, hence with a depth much closer to the reflectionless case of $U_{0}=-1$ than the examples in Figure 6b–d. The other parameter values are those of Figure 6. Notice that the emerging forbidden quasi-bands are anomalously narrow (essentially spikes), a kind of reflection comb. This pattern continues for larger *k*s, although we have not analyzed the eventual threshold $k_{rc}$ for which the comb-like comportment is lost (this will be the subject of a future contribution). In Figure 7, the average separation between instances of $|T_{N}|\approx 0$ is Δk=0.23.

Additionally, we mention that, even for hugely large but finite *N*s, typically the allowed quasi-bands are not characterized by perfect transmission. As nicely shown in [44], one can still have fluctuations from $|T_{N}{|}^{2}=1$. This is indeed seen in Figure 6 as the “dark regions” in the plots of $|T_{N}{|}^{2}$. On the other hand, for the allowed quasi-bands in Figure 7, we practically do not see a departure from $|T_{N}{|}^{2}\approx 1$. Thence, at least in this regard the array in Figure 7 already exhibits features of an actual infinite lattice.

## 5. Anomaly in the Lattice Transmission Induced by Asymmetric Basic Cells

The recurrence relations in Equation (1) allow one to construct a multitude of distinct symmetrical and asymmetrical building blocks (SBB and ABB). This should lead to a great diversity of scattering processes taking place in the associated arrays, consequently impacting their global transport features. In this last section, we shall exemplify such a type of phenomenology, contrasting symmetric and asymmetric cells. So, we consider lattices having building blocks formed by two barriers, left and right, whose centers are a distance, *d*, apart. Consequently, the BBs are spatially symmetric (asymmetric) if these two barriers are equal (different). We notice that the transport properties of lattices having, as a cell, a single asymmetric potential, like trapezoidal and triangular barriers, have already been discussed elsewhere (for an overview, see, e.g., [27]). However, as far as we know, the particular effect we characterize here is not directly comparable with the ones in these other studies.

**Figure 8:**
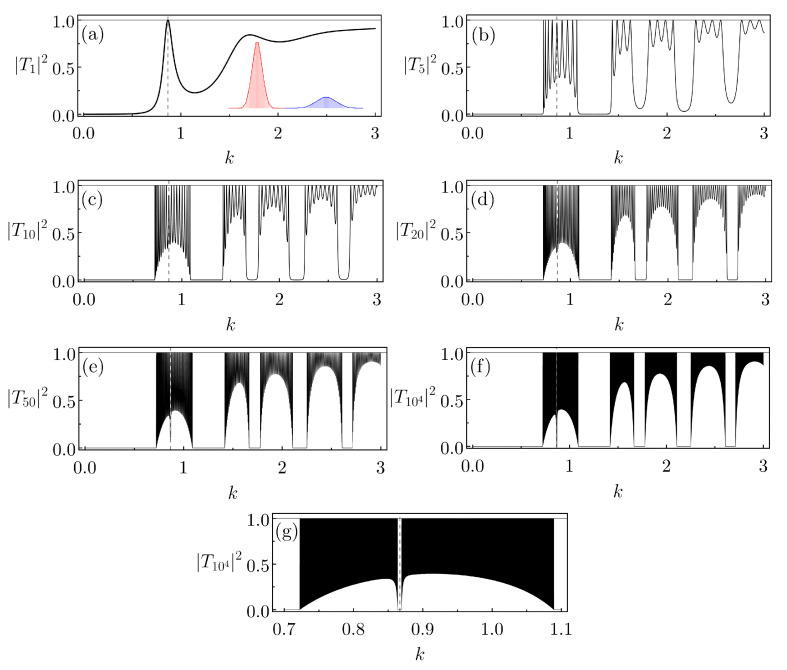
(**a**) For the Gaussian ABB discussed in the main text (and approximated by two CBB-IVs, inset) of parameters $U_{0}=4,\;\sigma =0.1$ (left, the red graphic in the inset) and $U_{0}=0.66,\;\sigma =0.6$ (right, the blue graphic in the inset) barriers, the resulting transmission probability, $|T_{1}{|}^{2}$, shown as a function of *k*. For the *N* finite periodic lattices, the corresponding $|T_{N}{|}^{2}\left(k\right)$ are displayed in (**b**) N=5, (**c**) N=10, (**d**) N=20, (**e**) N=50, (**f**) $N=10^{4}$. In all cases, the distance between successive cells (formed by two CBB-IVs) are equal to one. A blow up of (**f**) in a particular *k* interval emcopassing $k_{res}$ is shown in (**g**).

We start by supposing an ABB composed of two Gaussians of parameters $U_{0}=4,\;\sigma =0.1$ and $U_{0}=0.66,\;\sigma =0.6$, moreover, with d=1. The calculations are then approximated by our previous CBB-IVs (the comparison between the double Gaussians and the double CBB-IVs, as well as the parameters for the latter, are presented, respectively, in the inset of Figure 8a and in Table 2). The transmission probability through the ABB, denoted as $|T_{N=1}{|}^{2}$, is shown in Figure 8a. We observe that around k=0.9 there is a peak where $|T_{1}{|}^{2}=1$. In other words, $k_{res}\approx 0.9$ is a quasi-state resonance, allowing a 100% transmission through the ABB structure.

For the lattices formed by this ABB, we set the distance between the cells (constituted by two CBB-IVs) to be 1. In Figure 8b–f we depict the resulting $|T_{N}{|}^{2}$s versus *k* for *N* equal to 5, 10, 20, 50 and $10^{4}$. As expected, as *N* increases we observe the formation of quasi-band structures for the associated systems. Nonetheless, another effect also arises. The transmission probability for $k\approx k_{res}$ gradually shifts away from one, eventually vanishing for large *N*s. Figure 8g represents a blow-up of Figure 8f in a *k* interval about $k_{res}$. It highlights the emergence of a type of defect or *rip* in the allowed quasi-band corresponding to $k_{res}$. Hence, instead of permitting a full propagation along the array when the incident wave has $k\approx k_{res}$, the original quasi-bond state of the ABB cell turns into a trapped state of the finite lattice—provided *N* is large enough. Although not explicitly displayed here (but see below), we report that, for Gaussian SBBs, we have not observed the above-mentioned rips in the lattice bands around the cell $k_{res}$s.

**Figure 9:**
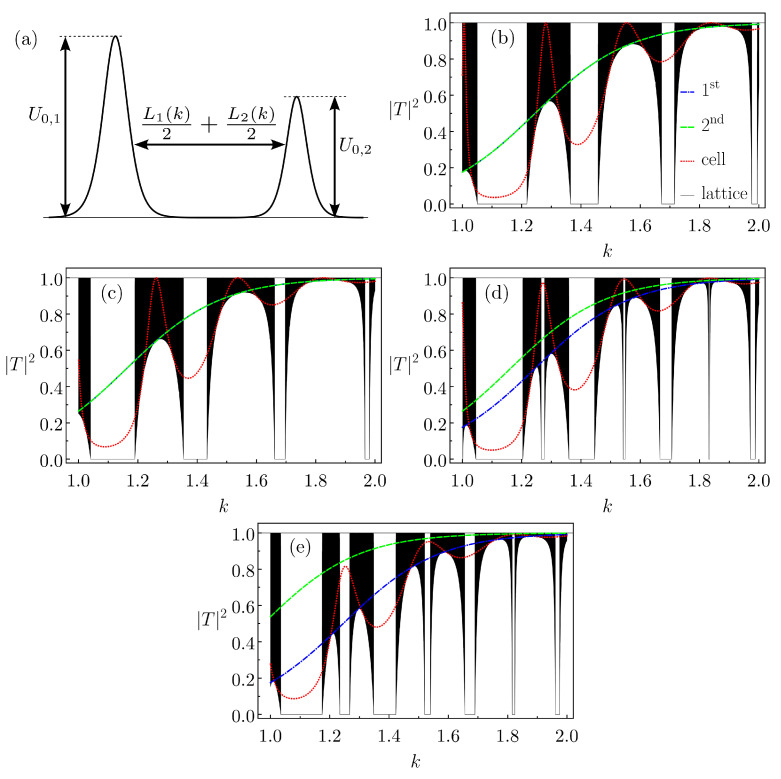
(**a**) Schematics of a cell formed by two Pöschl–Teller barriers, where the distance between them as a function of *k* is $L\left(k\right)=L_{1}\left(k\right)/2+L_{2}\left(k\right)/2$. The transmission probability of a single cell, $|T_{1}{\left(k\right)|}^{2}$, and of a lattice with $N=10^{4}$ cells, $|T_{N}{\left(k\right)|}^{2}$, are shown for the cases of (**b**) $U_{0,1}=U_{0,2}=0.9$, (**c**) $U_{0,1}=U_{0,2}=0.8$, (**d**) $U_{0,1}=0.9$, $U_{0,2}=0.8$, (**e**) $U_{0,1}=0.9$, $U_{0,2}=0.6$. In all cases, α=1 and c=5. The transmission probabilities for the isolated left and right barriers are also displayed.

To investigate if such a phenomenon is not only due to the particularities of Gaussians, we also suppose the Pöschl–Teller potential. To simplify the analysis, we set the same α=1 and c=5 for both cell barriers (recall that the centers are 2c=10 apart), defining $U_{0,1}$ (left) and $U_{0,2}$ (right) as their heights. Obviously, for SBB (ABB) we have $U_{0,1}=U_{0,2}$ ($U_{0,1}\neq U_{0,2}$). The calculations for $|T_{N}{|}^{2}$ follow the procedure in the previous section. We only mention that to obtain $R^{\left(\pm \right)}$ and *T* for the Pöschl–Teller SBB and ABB—from the recurrence relations in Equation (4)—we set $L\to L_{1}\left(k\right)/2+L_{2}\left(k\right)/2$ (refer to Figure 9a), with $L_{j}$ given by Equation (7). Figure 9b,c display $|T_{1}{\left(k\right)|}^{2}$ for SBBs with $U_{0,1}=U_{0,2}=0.9$ and $U_{0,1}=U_{0,2}=0.8$. Resonance $k_{res}$s are observed for both SBBs. The transmission probabilities for the related lattices with $N=10^{4}$ cells are also shown. Note that no defects (or rips) are identified in the allowed quasi-bands corresponding to these $k_{res}$s.

The situation is distinct for ABBs. In Figure 9d, we have similar plots, but now for a basic cell with $U_{0,1}=0.9$ and $U_{0,2}=0.8$. As in Figure 8, the defects do appear for the quasi-bands corresponding to the *k*s for which $|T_{1}{|}^{2}\approx 1$. For example, for k≈1.27,1.55,1.83 it reads $|T_{1}{|}^{2}\approx 0.97,0.99,1.00$ and we clearly see a kind of defect in the quasi-bands related to such *k*s. For an ABB with $U_{0,1}=0.9$ and $U_{0,2}=0.6$, the results are shown in Figure 9e, displaying a somehow similar phenomenology. However, for k≈1.25,1.53,1.82, then $|T_{1}{|}^{2}=0.82,0.95,0.99$. So, for this ABB the transmission probability local peaks tend to be smaller. As a consequence, the defects are more like a very narrow forbidden quasi-band than a rip.

The present analysis is also repeated for cells formed by two rectangular barriers in Appendix D. We find exactly the same type of behavior.

A natural question is why the defects arise only when the double barrier cells are asymmetric. This can be explained by considering the multiple scattering processes taking place within each cell and in between the different cells. First, the sole cell resonances, $k_{res}$s, are a direct consequence of the multiple scattering occurring between its left and right barriers. But if the cells are symmetric, once placed in an array, the full structure in fact can be viewed as a set of 2N equal barriers, and in a sense the identity of the individual double barrier cells is lost. Therefore, any emergent undulatory process is a consequence of the multiple scattering taking place for all the localized 2N potentials, each contributing at the same footing. Even if the distances between two cell barriers, *d*, and two consecutive cells, *L*, are distinct, this only introduces two length scales (so two characteristic phases), but the single barriers scattering coefficients are all the same along the array. Conversely, for asymmetric cells, effectively we have two distinct multiple scattering processes: that in each cell and that among the distinct cells along the whole lattice. Thus, in general, the $k_{res}$s are not simply washed out by organizing the isolated cells into a network. As a result, the corresponding quasi-state resonances must influence the full structure comportment, and by translational invariance symmetry yield lattice trapping states such that $|T_{N}\left(k_{res}\right){|}^{2}\approx 0$.

As a parallel, the Su–Schrieffer–Heeger (SSH) model [42,53,54], widely employed in the study of polymers such as polyacetylene, exhibits a similar gap opening behavior. This tight-binding model depicts a single spinless electron on an 1D lattice with two (distinct) site unit cells, simulating a lattice distorted by the Peierls instability [55,56]. Thus, the electrons have only one degree of freedom, hopping between sites. The gap opening is then caused by the difference between the interior and exterior hopping potentials, with the former (latter) referring to the dynamics within the unit cell (connecting adjacent unit cells). Hence, phenomenologically the two hopping potentials in the SSH correspond to the two distinct multiple scattering mechanisms unveiled in our work.

Finally, we shall mention that anomalous behavior related to the transport properties of a lattice often arises from asymmetrical configurations. For instance, numerical analysis of 1D photonic arrays has revealed that asymmetric defects significantly affects beam propagation [57]. Also, when considering non-periodic 1D lattices with momentum conservation, an asymmetric potential can induce normal heat conduction due to a finite-size effect, with such an anomaly emerging in the thermodynamic limit [58]. Analogous results have been observed in three-dimensional networks. As an example, we cite the shape memory of alloy cellular lattices. Micro-structural imperfections within the cells lead to spatial asymmetries, which significantly influence the mechanical response of the entire lattice [59].

## 6. Final Remarks and Conclusions

In the present work, we have analyzed the influence of the local features of certain basic cells on the transmission properties of finite, but long, periodic arrays. So, based on a framework developed in [27], we have developed a simple scheme to generate distinct building blocks (the cells) with different structures and to calculate their resulting reflection, $R^{\left(\pm \right)}$, and transmission, *T*, coefficients. It is worth mentioning that for the many distinct potentials (in particular those addressed here), which could be used to construct the cells, the proposed approach tends to be computationally more efficient than the potential slicing technique (based on rectangular barriers and the transfer matrix method). As case studies, we have discussed combinations of Gaussian and of Pöschl–Teller potentials, obtaining very good numerical results.

Importantly, we have unveiled certain anomalous behavior for transport in structures induced by their cells’ rather special characteristics. For lattices composed of Pöschl–Teller wells, when the isolated potentials have their parameter values close to the conditions of 100% transmission (a remarkable property of $U_{PT}$), the transport profile along the array resembles an unusual "reflection comb". In other words, $|T_{N}{|}^{2}$ is not one only for very determined values of *k*, when it practically vanishes.

A second anomalous effect was observed in periodic very long structures formed by double barrier cells. For asymmetric shapes, the allowed quasi-bands corresponding to the $k_{res}$s display a kind of defect, like a rip, which becomes narrower and narrower for $|T_{N}\left(k_{res}\right){|}^{2}$ closer and closer to 1. Nevertheless, such a phenomenon does not take place if the cells are spatially symmetric. The proper physical reasons for this have been discussed.

Since the segmentation of different continuous localized potentials into rectangular barriers is a common procedure to treat contexts like tunneling through gate oxides [60,61] and quantum dots [62], our protocol here could be an important new tool to deal with these same types of problems. Moreover, well-tailored CBBs could mimic relevant continuous potentials, e.g., approximating Lennard–Jones-like and Morse-like *U*s, which are often employed to describe inter-atomic effective interactions [63].

We finally mention that other possible applications for the present general method relate to looking for anomalous behavior in very promising systems (aimed at optical and thermal devices) like 1D photonic crystals [64,65,66] and low-dimensional solid structures such as nanotubes and nanowires [67].

## Figures and Tables

**Table 1:** Parameters employed to construct the CBBs, Figure 2a, that approximate the Gaussian barrier in Equation (5), with μ=0, $\sigma =\sqrt{2}/2$, $U_{0}=1$. The CBB-II,III,IV have a central rectangular barrier, with w=0.4 and $\tilde{U}=1$. The triangular (t) and trapezoidal ($\tilde{t}$) shapes to the left and right sides of the CBB centers are specular images of one another. So, only the values of the left Basic Shapes are indicated.

CBB	Basic Shape	*w*	$U_{a}$	$U_{b}$
I	t	1.5	0	1.15
II	t	1.3	0	1
III	t	0.9	0	0.15
	$\tilde{t}$	1.1	0.15	1
IV	t	0.6	0	0.07
	$\tilde{t}$	0.5	0.07	0.3
	$\tilde{t}$	0.9	0.3	1

**Table 2:** Parameters of the CBB-IVs in Figure 5a, displayed in the same way as in Table 1. For the rectangular Basic Shape $\tilde{U}=U_{0}$ and w=0.05,0.1,0.2,0.3, respectively, for σ=0.1,0.2,0.4,0.6.

Gaussian ($U_{0},\sigma$)	Basic Shape	*w*	$U_{a}$	$U_{b}$
(4,0.1)	t	0.08	0	0.3
	$\tilde{t}$	0.07	0.3	1.3
	$\tilde{t}$	0.125	1.3	4
(2,0.2)	t	0.16	0	0.15
	$\tilde{t}$	0.14	0.15	0.65
	$\tilde{t}$	0.25	0.65	2
(1,0.4)	t	0.35	0	0.09
	$\tilde{t}$	0.25	0.09	0.33
	$\tilde{t}$	0.5	0.33	1
(0.66,0.6)	t	0.5	0	0.05
	$\tilde{t}$	0.3	0.05	0.17
	$\tilde{t}$	0.85	0.17	0.66

**Figure A1:**
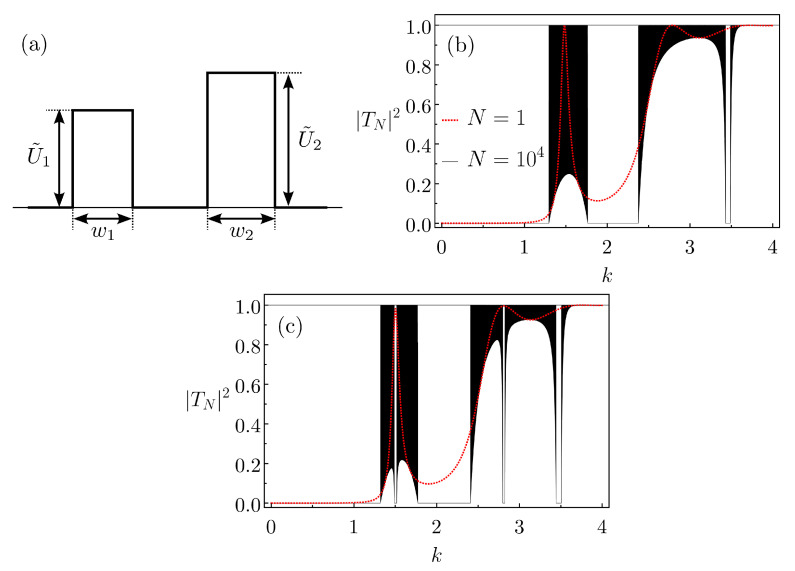
(**a**) Illustration of a building block formed by two rectangular barriers, with widths $w_{1},w_{2}$ and heights ${\tilde{U}}_{1},{\tilde{U}}_{2}$. Graph of the transmission probability in terms of the wavenumber *k*, when we consider (**b**) a symmetrical and (**c**) an asymmetrical cell. In (**b**), ${\tilde{U}}_{2}=2$, and in (**c**), ${\tilde{U}}_{1}=2.2$. The remain parameters are $w_{1}=w_{2}=1$, ${\tilde{U}}_{1}=2$ in both figures. The width between any two consecutive barriers is unitary.

## Data Availability

The data supporting this study’s findings are available from the corresponding author upon request.

## Abbreviations

The following abbreviations are used in this manuscript:

CBB	Composed building block
WM	Wronskian method
TM	Transfer matrix method
SBB	Symmetrical building block
ABB	Asymmetrical building block

## Appendix A. General *R* and *T* Coefficients

Assume a compact support potential, *U*, which is non-zero only in an interval a<x<b and whose usual quantum reflection and transmission coefficients are denoted by *r* and *t*. Considering a plane wave of wavenumber *k*, incoming from x<a (+) or x>b (−), we can define *R* and *T* as $r^{\left(\pm \right)}\left(k\right)=\exp\left[\pm 2\;i\;k\;c^{\left(\mp \right)}\right]\;R^{\left(\pm \right)}\left(k\right)$ and $t^{\left(\pm \right)}\left(k\right)=\exp\left[-i\;k\;\left(b-a\right)\right]\;T^{\left(\pm \right)}\left(k\right)$, with $c^{\left(+\right)}=b$ and $c^{\left(-\right)}=a$. In other words, the scattering amplitudes for a compact support potential can be written as a product of a complex function (*R* and *T*) and a phase related to the onset points of *U* [25]. Note that ${\left|r\right|}^{2}={\left|R\right|}^{2}$ and ${\left|t\right|}^{2}={\left|T\right|}^{2}$. In addition, for problems with time-reversal symmetry, $T^{\left(\pm \right)}=T$. Also, if *U* is symmetric, $R^{\left(\pm \right)}=R$.

When addressing a multiscattering problem, the exact Green’s function is expressed as a sum over all possible scattering paths, where each term is a product of *R* or *T* by an exponential involving the classic action. Since the paths already take into account the phases $\exp[\pm 2\;i\;k\;c^{\left(\mp \right)}]$ and exp[−ik(b−a)], one should use *R* and *T*, instead of *r* and *t*. This explains the usage of $R^{\left(\pm \right)}$ and *T* in Section 2.

## Appendix B. Rectangular, Trapezoidal and Triangular Barriers

Here we summarize the quantum amplitudes for rectangular, trapezoidal and triangular barriers, which are used to construct the CBBs discussed in the present work.

First, consider the symmetric rectangular barrier of width *w* and height $\tilde{U}$. Its scattering amplitudes are easily found in textbooks. They are given by

(A1) $$R_{rect}=-\frac{i\;\left(\kappa^{2}+k^{2}\right)\sinh\left[\kappa \;w\right]}{A(k)},\;T_{rect}=\frac{2\;\kappa \;k}{A(k)},$$

where $\kappa =\sqrt{2\tilde{U}-k^{2}}$ and $A\left(k\right)=2\;\kappa \;k\;\cosh\left[\kappa \;w\right]+i\;\left(\kappa^{2}-k^{2}\right)\sinh\left[\kappa \;w\right]$.

Suppose now the trapezoidal potential

(A2) $$\begin{array}{ccc} U(x) & = & \frac{\left(U_{b}-U_{a}\right)\;x+\left(U_{a}\;b-U_{b}\;a\right)}{\left(b-a\right)},\;\;a\leq x\leq b, \end{array}$$

and U(x)=0 otherwise. It is asymmetric since $U\left(a\right)=U_{a}$ and $U\left(b\right)=U_{b}$. From Equation (A2), we can also obtain a triangular barrier by setting either $U_{a}$ or $U_{b}$ to zero. Its reflection and transmission coefficients are analytically calculated (see, for example, [27]), so that

(A3) $$R_{trap}^{\left(\pm \right)}\left(k\right)=\frac{B^{\left(\pm \right)}\left(k\right)}{C(k)},\;T_{trap}\left(k\right)=-\frac{2\;\eta (k)}{\pi \;C(k)},$$

with

(A4) $$\alpha =2\frac{\left(U_{a}-U_{b}\right)}{\left(b-a\right)},\;\beta =2\frac{\left(U_{a}\;b-U_{b}\;a\right)}{\left(b-a\right)},\;y_{x}=\left[-x+\frac{\left(\beta -k^{2}\right)}{\alpha }\right]\;\alpha^{1/3},\;\eta \left(k\right)=\frac{i}{k}\;\alpha^{\frac{1}{3}},$$

and

(A5) $$\begin{array}{ccc} B^{\left(\pm \right)}\left(k\right) & = & \left[\mathrm{Ai}\left(y_{a}\right)\mp \eta \left(k\right)\;{\mathrm{Ai}}^{\prime }\left(y_{a}\right)\right]\;\left[\mathrm{Bi}\left(y_{b}\right)\mp \eta \left(k\right)\;{\mathrm{Bi}}^{\prime }\left(y_{b}\right)\right]\\ & & -\left[\mathrm{Ai}\left(y_{b}\right)\mp \eta \left(k\right)\;{\mathrm{Ai}}^{\prime }\left(y_{b}\right)\right]\;\left[\mathrm{Bi}\left(y_{a}\right)\mp \eta \left(k\right)\;{\mathrm{Bi}}^{\prime }\left(y_{a}\right)\right],\\ C(k) & = & \left[\mathrm{Ai}\left(y_{a}\right)+\eta \left(k\right)\;{\mathrm{Ai}}^{\prime }\left(y_{a}\right)\right]\;\left[\mathrm{Bi}\left(y_{b}\right)-\eta \left(k\right)\;{\mathrm{Bi}}^{\prime }\left(y_{b}\right)\right]\\ & & -\left[\mathrm{Ai}\left(y_{b}\right)-\eta \left(k\right)\;{\mathrm{Ai}}^{\prime }\left(y_{b}\right)\right]\;\left[\mathrm{Bi}\left(y_{a}\right)+\eta \left(k\right)\;{\mathrm{Bi}}^{\prime }\left(y_{a}\right)\right]. \end{array}$$

Above, Ai(y), ${\mathrm{Ai}}^{\prime }\left(y\right)$ (Bi(y), ${\mathrm{Bi}}^{\prime }\left(y\right)$) represent, respectively, the Airy function of the first (second) kind and its derivative with respect to *y*.

## Appendix C. Scattering Coefficients for the *U_PT_*(*x*) = *U*_0_/cosh^2^[*α x*] Potential

For the $U_{PT}$ potential of Equation (6), in Equation (4) one uses $R^{\left(\pm \right)}=R_{PT}\;\exp\left[i\;k\;\phi \left(K\right)\right]$ and $T=T_{PT}\;\exp\left[i\;k\;\phi \left(K\right)\right]$ for (see [37] and the references therein)

(A6) $$\begin{array}{ccc} T_{PT} & = & \frac{k}{i\alpha }\frac{\Gamma (-ik/\alpha -s)\Gamma (-ik/\alpha +s+1)}{\Gamma (-ik/\alpha +1)\Gamma (-ik/\alpha +1)},\\ R_{PT} & = & \frac{\Gamma (-ik/\alpha -s)\Gamma (-ik/\alpha +s+1)\Gamma (ik/\alpha )}{\Gamma (-s)\Gamma (s+1)\Gamma (-ik/\alpha )}. \end{array}$$

Here, Γ(·) is the gamma function, $\epsilon =U_{0}/E=2U_{0}/k^{2}$ and $s=(-1+\sqrt{1-8U_{0}/\alpha^{2}})/2$. A phase ϕ(k) multiplying the reflection and transmission amplitudes of the basic cells is necessary [37] when the potential is not of compact support. For the Pöschl–Teller potential, it reads

(A7) $$\phi \left(k\right)=\frac{1}{\alpha }\left(2\sqrt{\epsilon }\ln\left[\sqrt{\epsilon }+1\right]+\left(1-\sqrt{\epsilon }\right)\ln[|\epsilon -1|]\right).$$

## Appendix D. Double Rectangular Barrier Cells and Anomalous Behavior

Consider the building block formed by two rectangular barriers, as illustrated in Figure A1. If the widths $w_{1}$ and $w_{2}$ and heights ${\tilde{U}}_{1}$ and ${\tilde{U}}_{2}$ are the same (different), we have an SBB (ABB). The reflection and transmission coefficients for a single rectangular potential are given in Appendix B. Hence, the coefficients for the double barrier cell are simply determined from the recurrence relations in Equation (1).

Assume $w_{1}=w_{2}=1$, ${\tilde{U}}_{1}={\tilde{U}}_{2}=2$ and an unitary separation between any two successive cells for a lattice with $N=10^{4}$. For this case, in Figure A1b we show the transmission probabilities as a function of *k* for N=1 and $N=10^{4}$. Note that, for this symmetric cell, the $k_{res}$s do not influence the lattice quasi-band structure. On the other hand, for an ABB cell with the exact same parameters, unless for ${\tilde{U}}_{2}=2.2$, the profiles of $|T_{1}{\left(k\right)|}^{2}$ and $|T_{10^{4}}{\left(k\right)|}^{2}$ are depicted in Figure A1c. Now, we clearly observe rip-like defects (around the cell $k_{res}$s) in the lattice-allowed quasi-bands. These corroborate the results in Section 5
